# Fusogenic liposome-enhanced cytosolic delivery of magnetic nanoparticles†

**DOI:** 10.1039/d1ra03094a

**Published:** 2021-11-04

**Authors:** Fang Chen, Minjuan Bian, Michael Nahmou, David Myung, Jeffrey L. Goldberg

**Affiliations:** Department of Ophthalmology, Spencer Center for Vision Research, Byers Eye Institute at Stanford University Palo Alto CA 94304 USA djmyung@stanford.edu; VA Palo Alto Health Care System Palo Alto CA 94304 USA; Department of Chemical Engineering, Stanford University Stanford CA 94305 USA

## Abstract

Magnetic nanoparticles (MNPs) are widely used in cell sorting, organelle selection, drug delivery, cell delivery, and cell tracking applications. However, organelle manipulation in living cells has been limited due to the endocytic uptake and sequestration of MNPs. Here, we introduce a method for modifying MNPs with fusogenic liposomes that facilitate MNP passage directly into the cytosol. MNPs were enclosed in fusogenic liposomes that exhibit a core–shell structure under a transmission electron microscope (TEM). The lipid-to-MNP ratio was optimized for one layer of liposome coating around each MNP, so that MNPs were delivered to the cytosol without endosomal or liposomal coatings. After incubation with the retinal pigment epithelial cell line ARPE-19, single-layer liposome-coated MNPs exhibited the highest MNP delivery efficiency. Although uncoated MNPs are taken up through endocytosis, less than 15% of the fusogenic liposome-coated MNPs co-localized with early endosomes. MNPs delivered by fusogenic liposomes showed cytosolic localization early on and increased lysosomal localization at later time points. The movement of intracellular MNPs could be manipulated with an external magnet to estimate cytosolic viscosity. Bypassing endocytosis in this way allowed efficient delivery of MNPs to the cytosol, potentially allowing for the targeting of specific organelles and controlling their motion in living cells.

## Abstract 2

Fusogenic liposomes facilitate MNPs passage into the cytosol and enable direct contact between MNPs and organelles other than endosomes.

## Graphical abstract

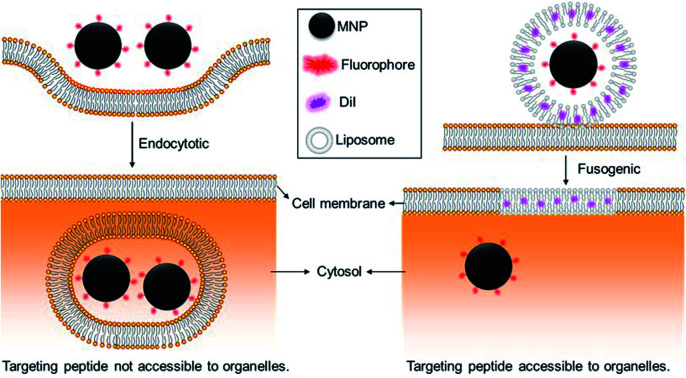

## Introduction

Labeling cells with nanoparticles has broad applications in cell imaging, tracking, manipulation, and drug delivery.^1–4^ Iron oxide nanoparticles have been extensively studied for their magnetic properties and wide applications in biology and life science.^5,6^ First, they are widely used to track cells *in vivo* using magnetic resonance imaging (MRI)^7^ and as a contrast agent for the emerging imaging modality known as magnetic particle imaging.^8^ Iron oxide nanoparticles can be easily surface-modified with various targeting ligands.^9,10^ By taking advantage of their paramagnetism, iron oxide nanoparticles have been used in targeted drug delivery^11,12^ and commercialized to purify biological samples such as specific cell types, organelles (from cell lysates), and macromolecules.^13–15^ Iron oxide nanoparticles have also shown great promise in the delivery of cell transplant therapies *in vivo*, which addresses localization, retention, and integration, thereby greatly increasing therapeutic potency and efficacy.^16–21^ In addition, magnetic particles have also been used to measure the intracellular viscosity of cells.^22^

An emerging and exciting application of iron oxide nanoparticles is to manipulate the localization of specific organelles in living cells in order to understand the effect of their distribution and movement on basic cell functions such as growth, differentiation, and homeostasis. For example, Steketee *et al.* functionalized magnetic nanoparticles (MNPs) with tropomyosin receptor kinase B (TrkB) agonist antibodies and used them to label primary neurons.^13^ The functionalized MNPs bound to TrkB on the cell membrane, activated TrkB-dependent signaling, and produced MNP-labeled signaling endosomes when MNPs were endocytosed by primary neurons. Manipulating MNP-labeled signaling endosomes with a magnetic field altered growth cone motility and halted neurite growth in both peripheral and central nervous system neurons, demonstrating that signaling endosome localization in the growth cone regulates motility and neurite growth.^13^

Most nanoparticles can enter and thereby label cells *via* simple co-incubation with nanoparticle-containing cell media in the absence of any transfection reagents.^23,24^ Nanoparticle uptake is mediated by endocytosis, which is the *de novo* production of internal membrane-bound structures derived from the plasma membrane lipid bilayer.^25^ Endocytosed nanoparticles are entrapped by the plasma membrane, which prevents their direct contact with cytosol and organelles with the exception of endosomal pathway organelles and ultimately lysosomes.^26^ Although endocytosed nanoparticles can indicate cellular localization, they do not enable the binding or subsequent delivery of therapeutics to specific intracellular compartments or other organelles. If there were no alternatives to the endocytic trafficking route, the potential usefulness of nanoparticles as intracellular probes and therapeutic agents would be limited, because the particles would never participate in any cytosolic or organelle-based events, except for those occurring within the endosomes to which they were confined.^27^ Hence, bypassing the endocytic uptake of nanoparticles is essential to improve intracellular cytosolic delivery and organelle manipulation.

**Fig. 1:**
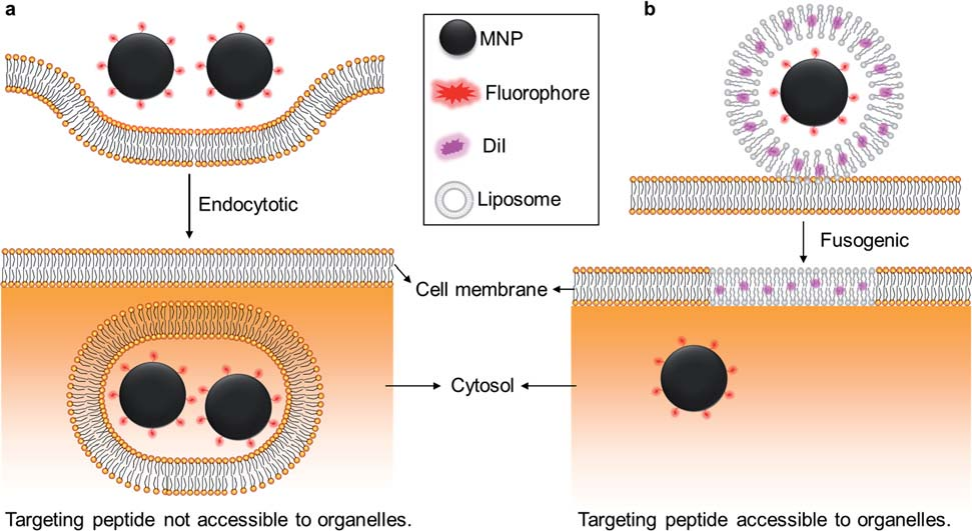
Schematic illustrations of (a) endocytic and (b) fusogenic cell uptake of magnetic nanoparticles (MNPs). The fusogenic pathway allows the surface of delivered MNPs to contact the cytosol and intracellular organelles directly.

Indeed, understanding the effect of the distribution and movement of other organelles such as mitochondria, ribosomes, and centrioles on cell biology remains an unmet need in basic science and translational drug delivery. This could be addressed through binding magnetic nanoparticles to these other specific organelles. While targeting organelles with MNPs has been realized by antibody conjugation, techniques to bypass the endocytic uptake of MNPs have not been realized.^28^ Here, we demonstrate a novel technique using cell membrane-fusogenic liposomes, which have previously facilitated direct cytosolic delivery of proteins,^29,30^ polymeric nanoparticles,^26^ and silica nanoparticles.^31^ Using this fusogenic liposome-enhanced cytosolic delivery, we demonstrate bypass of endocytosis and delivery of iron oxide nanoparticles directly into the cytosol (Fig. 1) and test the cytosolic magnetic nanoparticles' movement and distribution in living cells.

## Experimental

### Materials

All lipids were purchased from Avanti Polar Lipids, including 1,2-dimyristoyl-*sn*-glycero-3-phosphocholine (DMPC, 850345C), 1,2-distearoyl-*sn*-glycero-3-phosphoethanolamine-*N*-[methoxy(polyethylene glycol)-2000] (ammonium salt) (DSPE-PEG, 880120C), 1,2-distearoyl-*sn*-glycero-3-phosphoethanolamine-*N*-[amino(polyethylene glycol)-2000]-*N*-(cyanine 5) (DSPE-PEG-CY5, 810891C), and 1,2-dioleoyl-3-trimethylammonium-propane (DOTAP, 890890C). 1,1′-dioctadecyl-3,3,3′,3′-tetramethylindocarbocyanine perchlorate (DiI) and Alexa Fluor™ 647 Succinimidyl Ester were purchased from Thermo Fisher Scientific. Iron oxide nanoparticles (Amine Super Mag Magnetic Beads, SA0052) were obtained from Ocean NanoTech. Dimethyl sulfoxide (DMSO) was purchased from Sigma-Aldrich. Gadolinium triacetate based staining solution was purchased from Ted Pella (Prod # 14985, ∼10% gadolinium triacetate), and the staining solution was used without any dilution. CellLight® early endosome-GFP, CellLight® lysosome-GFP, and CellLight® plasma membrane-GFP were from Thermo Fisher Scientific.

### Dye conjugation onto MNPs

MNPs were first conjugated with Alexa Fluor™ 647 through the succinimidyl ester chemistry. The stock buffer of MNPs was removed through the magnetic separation with the aid of LS Column placed in a QuadroMACS separator (Miltenyi Biotec). MNPs were then washed and collected in certain volume of PBS (pH 7.4) to get a final concentration of 1 mg mL^−1^ MNPs. Alexa Fluor™ 647 (100 μg) was dissolved in 20 μL of DMSO and then transferred to 10 mL of MNPs suspension. The mixture was allowed to react at 4 °C in dark overnight. The MNPs were then purified through magnetic separation and washed with PBS. The dye conjugated MNPs were resuspended in PBS at 1 mg mL^−1^ and stored at 4 °C in dark.

### Liposome synthesis and liposomal coating on MNPs

The liposome coating was prepared from DMPC, DSPE-PEG, and DOTAP at the molar ratio of 76.2 : 3.8 : 20.^31^ All lipids were dissolved in chloroform at 10 mg mL^−1^. DiI was dissolved in ethanol at 1.25 mg mL^−1^. The lipid films were prepared by evaporating the organic solvents, with 72.55 μL of DMPC, 15.16 μL of DSPE-PEG or DSPE-PEG-Cy5, 19.63 μL of DOTAP, and 20 μL DiI. For the liposome synthesis, the above lipid was hydrated with 1 mL PBS buffer by pipetting and stirring at 40 °C in the dark for 10 minutes. The mixtures were then extruded through a 200 nm polycarbonate membrane 20 times. The formed liposomes were stored at 4 °C in dark. For the liposomal coating on MNPs, the above lipid mixture was added to 0.5 mL, 1 mL, 2 mL, 4 mL, or 8 mL of MNPs suspension to make the initial lipid-to-MNPs ratios at 2.2, 1.1, 0.55, 0.28, or 0.14. The mixtures were stirred at 40 °C in the dark for 10 minutes. The mixtures were then extruded through a 200 nm polycarbonate membrane 20 times. Magnetic separation was used to remove hollow liposomes. The liposome-coated MNPs were restored in PBS and stored at 4 °C in dark for late use.

### Characterizations

Mass concentrations of MNPs and lipids were determined by absorbance and fluorescence, respectively (Spark plate reader). The size and zeta-potential of nanoparticles were measured by dynamic light scattering (Zetasizer ZS90, Malvern Instruments). Structural morphology was visualized by an FEI Tecnai G2 F20 X-TWIN Transmission Electron Microscope. TEM samples were prepared by dropping 5 μL of the sample on the TEM grid, drying off excess solvent after 1 min, and dropping 5 μL of gadolinium triacetate based staining solution for negative staining.

### Calculation of lipids per MNP

The calculation of the number of lipids per MNP was based on the following two assumptions: first, the MNPs were assumed to be perfect and identical spheres; second, the liposomal coating was assumed to be even and identical on every MNP. Particle concentrations of MNPs were calculated according to eqn (1), which was based on the first assumption that the MNPs were perfect and identical spheres with a radius of *r*. Based on the second assumption, dividing the weight ratio of coated lipids-to-MNPs by MNPs particle concentrations gave the weight of lipids per MNP. The total weight of lipids per MNP was then proportioned to each type of lipids and divided by the weight of a single lipid molecule, and the sum of the number of each lipid molecule was the lipid number per MNP.1
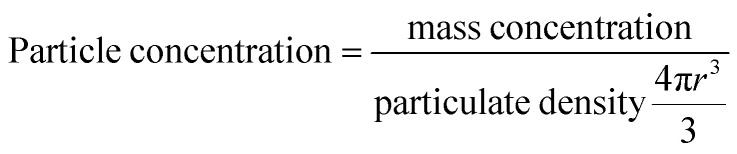


The mass concentrations of MNPs and lipids as well as the weight ratio between coated lipids and MNPs were experimentally determined by the absorbance and fluorescence. The number of lipid molecules per liposome (*n*) was calculated according to eqn (2), where *A* is the mean head group area of lipid molecules, *D* is the outer diameter of the liposome, and *t* is the thickness of the lipid bilayer.2
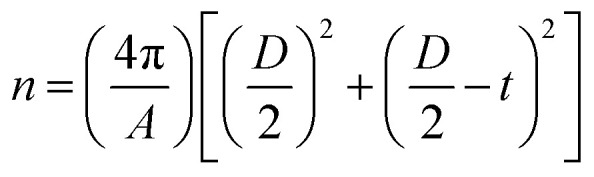


#### Cell culture

Human retinal pigment epithelial cell line ARPE-19 was obtained from American Type Culture Collection (ATCC, USA) and cultured in DMEM:F12 (Thermo Fisher Scientific, USA) supplemented with 10% fetal bovine serum (FBS), 50 μg mL^−1^ streptomycin, and 50 U mL^−1^ penicillin (Thermo Fisher Scientific, USA).

### Cell treatment with liposome coated MNPs

Liposome-coated MNPs were suspended in cell culture medium with MNP at the concentration of 100 μg mL^−1^ without any transfection agents. Cells were treated with liposome-coated MNPs or bare MNPs for 10 min at 37 °C in a humidified CO_2_ incubator. Cells were then washed with a fresh culture medium to remove free nanoparticles and cultured for the indicated duration.

### Cell staining and confocal imaging

CellLight® early endosome-GFP targeting Rab5a, CellLight® lysosome-GFP targeting Lamp1, and CellLight® plasma membrane-GFP targeting the myristolyation/palmitoylation sequence from Lck tyrosine kinase were used to label early endosome, lysosome, and plasma membrane, respectively. ARPE-19 cells were incubated with different CellLight® reagents for 24 h according to the manufacturer's instructions. Cells were fixed with 4% paraformaldehyde (PFA) for 10 min at room temperature at 0 h, 4 h, and 24 h after administration of liposome-coated MNPs, and cells were then washed twice and maintained in PBS. The fluorescence signals were acquired under a confocal microscope (LSM 880, Carl Zeiss, Germany). Measurement of identified DiI, Alexa Fluor 647, early endosome-GFP, and lysosome-GFP positive objects were collected by Volocity (Quorum Technologies, Canada). The fraction of free MNPs, MNPs touching early endosome, MNPs touching lysosome, liposome touching early endosome, and liposome touching lysosome were calculated and underwent statistical analysis.

### TEM cell sample preparation and imaging

Cells were detached, pelleted, fixed in 1.5 mL pre-warmed 2% glutaraldehyde (8% stock-EM grade) and 4% *p*-formaldehyde in 0.1 M HEPES, PO_4_, or sodium cacodylate buffer (pH ∼7.2) for 20 minutes at room temperature, and then moved to 4 °C for at least 40 minutes. The cells were then post-fixed with 1% osmium tetroxide with gentle rotation for 1 hour. The samples were then washed thrice in double-distilled water and counterstained with 1% uranyl acetate for 2 hours. The cells were then dehydrated with a series of ethanol at 50% and 70% for 5 minutes each, 95% for 10 minutes, and twice in 100% for 15 minutes. After that, the samples were changed to acetonitrile for 15 minutes. Finally, for epon (epoxy resin) infiltration, the samples were changed to 1 : 1 epon/acetonitrile for 1 hour, then 2 : 1 epon/acetonitrile for 1 hour, and finally 100% epon for 1 hour. The samples were then changed to fresh epon, polymerized at 65 °C. The samples were then cut, mounted on copper grids, and imaged under a TEM (JEOL JEM1400 – TEM1400) at 120 kV.

### Statistical analysis

All results were expressed as means ± standard error of the mean (S.E.M.). The statistical analysis was performed using one-way ANOVA followed by Tukey's multiple comparisons test (GraphPad Prism 8.4.1). Statistical significance was defined by *p* <0.05 and *p* <0.01.

## Results and discussion

The average diameter of the MNPs before any treatment was 61 ± 11 nm (*n* = 500) according to the TEM imaging. The size distribution is shown in ESI Fig. S1.† Before coating MNPs with liposomes, MNPs were conjugated with Alexa Fluor 647 *via N*-hydroxysuccinimide ester chemistry. The dye conjugation increased the hydrodynamic size of MNPs from 128 nm to 139 nm and kept the polydispersity index (PDI) within 0.2, which indicates a small size distribution of the MNPs before and after dye conjugation. It is reasonable that the TEM size of MNPs was smaller than that measured by DLS because DLS measures the hydrodynamic diameter, which is usually larger than the real size of nanoparticles. Of note, the size distributions of the dye-conjugated MNPs were consistent after 17 month storage in dark at 4 °C (ESI Fig. S2a–c†). An increase in the zeta potential of the MNPs from −20 to −7 mV indicated successful dye conjugation. The surface charge increased to −2 mV after 17 month storage in dark at 4 °C (ESI Fig. S2d†). The dye-conjugated MNPs were then mixed with lipids and co-extruded through a 200 nm polycarbonate membrane. The MNPs were successfully coated by liposomes and the coating was tunable by changing the initial lipid-to-MNP ratio, measured as total weight of lipids to weight of MNPs.

**Fig. 2:**
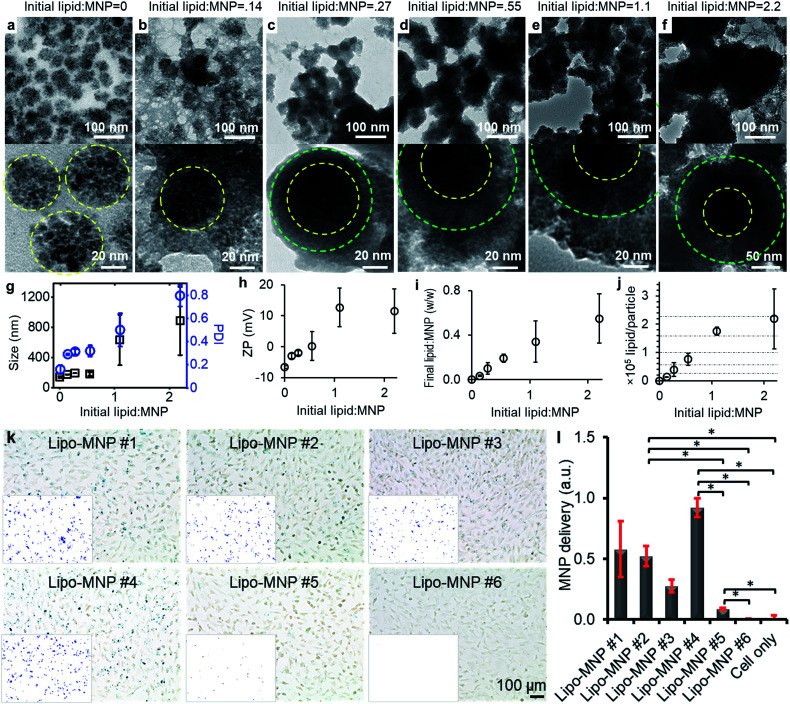
Effects of liposome coating on morphology, surface property, and cell uptake of MNPs. (a–f) TEM images of (a) bare MNPs that were imaged before extruding the liposome on MNPs (initial lipid-to-MNP ratio at 0) and liposome-coated MNPs with initial lipid-to-MNP ratios at (b) 0.14, (c) 0.28, (d) 0.55, (e) 1.1, and (f) 2.2 after extruding the liposome on MNPs. The yellow and green dashed curves in the high magnification TEM images outline the MNPs and the liposome coating respectively. (g) Hydrodynamic size and polydispersity of size distribution was determined by DLS. Both size and polydispersity of size distribution increased with the initial lipid-to-MNP weight ratio. (h) Zeta potential of MNPs increased with liposome coating. (i) The lipid-to-MNP of the product was positively dependent on the initial lipid-to-MNPs weight ratio. (j) Theoretical number of lipids on each MNP when all MNPs were coated evenly by liposomes. The dashed lines from bottom to top indicate numbers of lipids that are required to form 1, 2, 3, 4, and 5 layers of liposomes on a nanoparticle with diameter of 50 nm (minimal inner diameter of liposome according to the TEM size of MNP). (k) Light microscope images of iron stained ARPE-19 cells treated with liposome-coated MNPs with different initial lipid-to-MNP ratios. The insets show only the blue pixels in the area for a better visualization. (l) Quantification of MNP delivery efficiency into ARPE-19 cells with different liposome coatings. (*p <0.05, 2-tail homoscedastic Student's *t*-test).

First, we studied the effects of varying this ratio on the morphology and surface properties of the products. Fig. 2a–f shows transmission electron microscope (TEM) images of the products with initial lipid-to-MNP ratios of 0, 0.14, 0.28, 0.55, 1.1, and 2.2. The diameter of bare MNPs was approximately 50 nm, and they showed clusters of nanoparticle-like morphologies (Fig. 2a). Noticeably, when the initial lipid-to-MNP ratio was as low as 0.14, the features of bare MNPs were still detectable, and the core–shell structure was not obvious (Fig. 2b). However, these bare MNP features were replaced by a core–shell structure after liposomal coating as lipid content rose (Fig. 2c–f), indicating successful liposomal coating on MNPs. Therefore, it is critical to keep the initial lipid-to-MNP ratio higher than 0.14. High magnification TEM images showed that the shell became darker and thicker when more lipids were added during fabrication (Fig. 2c–f).

The average hydrodynamic diameters of the products by dynamic light scattering (DLS) analysis also showed a similar trend (Fig. 2g). The sample with an initial lipid-to-MNP ratio at 0 was the same as the dye-conjugated MNPs. Its average hydrodynamic diameter was approximately 140 nm. The filtration membrane used had a 200 nm pore size, so the liposome size was expected to be around 200 nm. Indeed, the average sizes of liposome-coated MNPs were 175, 192, and 198 nm when the initial lipid-to-MNP ratios were 0.14, 0.28, and 0.55, respectively. Therefore, each liposome could contain only one MNP. When the initial lipid-to-MNP ratio was 1.1 and 2.2, the average sizes were 631 nm (PDI = 0.501) and 884 nm (PDI = 0.796), indicating polydisperse size distribution, which is likely due to the aggregation of liposome-coated MNPs.

The fusogenic liposomes were made with DMPC (zwitterionic, neutral), DSPE-PEG (anionic, −1 charge), and DOTAP (cationic, +1 charge) lipids at a molar ratio of 76.2 : 3.8 : 20. Therefore, the coating with liposome was expected to increase the zeta potential of MNPs, and with more coatings leading to a more positive surface charge. Indeed, liposomal coating increased the zeta potential of MNPs, which turned from negative to positive when the initial lipid-to-MNP ratio increased from 0.28 to 0.55 (Fig. 2h). The initial lipid-to-MNP ratio also affected the number of liposomal layers coating the MNPs. Content of MNPs and coated lipids in the products were quantified based on their fluorescent signals after magnetic separation of (coated or uncoated) MNPs away from any residual free lipid and liposomes without MNPs. The liposome-to-MNP weight ratios increased as the initial lipid-to-MNP ratio increased. Thus, more liposomes coated the MNPs when more lipid was added, without reaching a plateau, consistent with adding additional layers or shells on average (Fig. 2i). The liposome-to-MNP weight ratios were converted to the number of lipids per MNP as described in ESI.† Theoretically, there were roughly 0, 14 000, 39 000, 76 000, 176 000, and 219 000 lipid molecules surrounding each MNP when the initial lipid-to-MNP (w/w) were 0, 0.14, 0.28, 0.55, 1.1, and 2.2, respectively. The number of lipid molecules per liposome layer was calculated using a reported method (ESI†).^32^ Approximately, there were 1, 2, 4, and 4 layers of liposome coated on the MNPs when the initial lipid-to-MNP ratio was 0.28, 0.55, 1.1, and 2.2, respectively. Less than 1 layer of liposome was coated on the MNPs on average when the initial lipid-to-MNP ratio was 0.14, consistent with TEM results (Fig. 2j).

Of note, these products showed different MNP delivery efficiencies in ARPE-19 cells. Fig. 2k shows microscope images of iron-stained ARPE-19 cells treated with liposome-coated MNPs with different initial lipid-to-MNP ratios. The presence of blue staining in the images indicates increasing iron content of the MNPs taken up by ARPE-19 cells. Liposome-coating increased the amount of MNPs in ARPE-19 cells, which suggests that the MNP delivery efficiency to ARPE-19 cells was increased by liposome coating. Fig. 2i showed the quantification of MNP delivery into ARPE-19 cells based on iron staining. There was no significant difference in iron staining between ARPE-19 cells only and cells treated with bare MNPs. Although bare MNPs could be endocytosed or pinocytosed by cells when cells were incubated with MNPs for hours,^33,34^ the short incubation time—10 minutes—in our experiments may explain why there were negligible MNPs detected in ARPE-19 cells treated with bare MNPs.

MNP delivery was the highest when coated with liposomes at an initial lipid-to-MNP ratio of 0.28, yielding theoretically one layer of liposome coating on MNPs (Fig. 2j and l). One layer of liposome coating is vital to facilitate direct contact between the delivered MNP and cytosol because MNPs will have no liposome coating once this liposome layer is fused with the cell membrane (Fig. 2b). Therefore, an initial lipid-to-MNP ratio of 0.28 is optimal to achieve a high MNP delivery efficiency into the cytosol. Of note, when the initial lipids-to-MNP ratio was higher than 0.55, the products showed a positive surface charge. Although the positive surface charge could increase the affinity between the products and cells, these products also showed a high degree of aggregation, which could prevent the fusion process of the fusogenic liposome and lead to decreased MNP delivery efficiency.

In light of these data, the lipid-to-MNP ratio of 0.28 was adopted for all subsequent studies. First, we examined the stability of the fluorescence signals and found that the DiI and Alexa Fluor 647 fluorescence signals of liposome-coated MNPs decreased to 48% and 62% respectively after the nanoparticles were stored for 17 months at 4 °C in dark. For comparison, the Alexa Fluor 647 fluorescence signal of the bare MNPs decreased to 34% of the original signal under the same storage conditions. Thus, the liposome coating protected the fluorescence signal of Alexa Fluor 647 conjugated MNPs, possibly by inhibiting contact between reactive radicals and the Alexa Fluor 647.^35^ The average DLS size decreased from 192 nm to 165 nm, accompanying a decreased PDI from 0.312 to 0.204 after 17 month storage. The surface charge of this product decreased slightly from −2 mV to −3 mV after 17 month storage.

The retinal pigment epithelium (RPE) plays key roles in retinal homeostasis, such as regulating the transport of nutrients and waste products, light absorption to protect photoreceptors from photo-oxidation, and phagocytosis of photoreceptor outer segment membranes. Degeneration or dysfunction of RPE have been reported to be closely related to the onset and progression of age-related macular disease (AMD) and inherited retinal dystrophies such as Stargardt's disease and retinitis pigmentosa. ARPE-19 cells are the most commonly used RPE cell line. We then investigated the cellular uptake and intracellular trafficking of liposome-coated MNPs in ARPE-19 cells. Of note, although DiI was not chemically conjugated onto the liposome, its fluorescence colocalized with the chemically conjugated dye (ESI S3†). Therefore, the DiI can signal the presence of the liposome. Alexa Fluor 647-conjugated MNPs coated with DiI-labeled liposome were applied to ARPE-10 cells with different incubation times (10 min, 4 h, or 24 h); distribution of liposome and MNPs indicated the best separation of liposome and MNPs with 10 min incubation (ESI Fig. S4†); therefore, the administration of liposome-coated MNPs to ARPE-19 cells with 10 min incubation was used for the following experiments. In order to examine whether the uptake of liposome-coated MNPs is membrane fusion dependent, ARPE-19 cells were treated with membrane fusion inhibitor enfuvirtide at 1 μM, 10 μM, and 100 μM for 1 h prior to 10 min incubation of liposome coated MNPs administration. The uptake of liposomes and MNPs was nearly completely inhibited with 100 μM treatment of enfuvirtide, indicating the uptake of liposome-coated MNPs is membrane fusion dependent (ESI Fig. S5†).

**Fig. 3:**
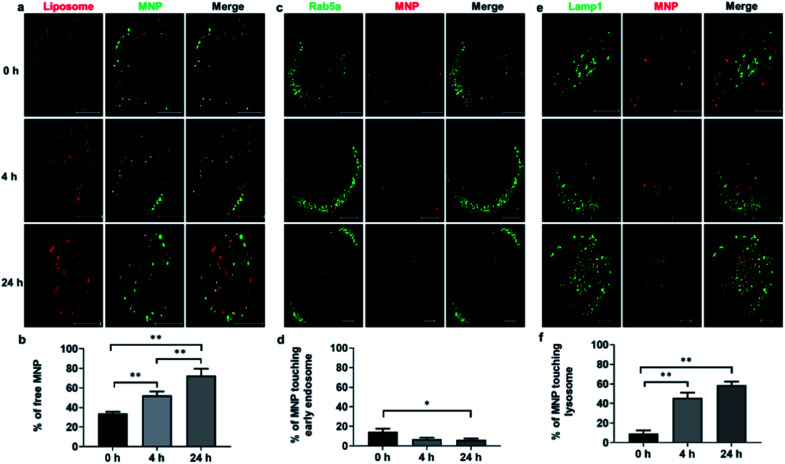
Intracellular distribution of liposome-coated MNPs after incubation with ARPE19 cells. CellLight® early endosome-GFP targeting Rab5a or CellLight® lysosome-GFP targeting Lamp1 was incubated with ARPE19 cells for 24 h, after which Alexa Fluor 647 conjugated MNPs coated with DiI labeled liposome were then administrated to ARPE-19 cells for 10 min. Cells were washed with PBS and fixed with 4% paraformaldehyde (PFA) for 10 min at room temperature at 0 h, 4 h and 24 h after incubation with liposome coated MNPs. DiI and MNP signals (a), early endosome-GFP and DiI (c) or lysosome-GFP and DiI signals (e) were acquired by confocal microscopy. Measurement of identified DiI, Alexa Fluor 647, early endosome-GFP and lysosome-GFP positive pixels were quantified with Volocity. The fractions of free MNPs (b), in which Alexa Fluor 647 signals didn't colocalize with liposomal DiI, Alexa Fluor 647 signals touching early endosome-GFP (d) or lysosome-GFP (f) were calculated and analyzed by one-way ANOVA followed by Tukey's multiple comparisons test (*p <0.05, **p <0.01, *n* = 8–15 cells).

The intracellular distribution of liposomes (DiI) and MNPs (Alexa Fluor 647) were monitored at 0 h, 4 h, and 24 h (Fig. 3a). Quantification of the fraction of free MNPs released from liposomes, defined as MNPs not colocalized with liposomes under fluorescence light microscopy, showed a significant increase over time, from 34.0% at 0 h to 72.7% at 24 h (Fig. 3b), illustrating the de-association of MNPs from liposomes after fusogenic entry into the cytoplasm. To test whether liposome coating affects endocytic entry, the otherwise default uptake pathway of MNPs,^25^ CellLight® early endosome-targeting Rab5a-GFP was used to label early endosomes and visualized with confocal imaging at 0 h, 4 h, and 24 h after 10 min incubation with liposome-coated MNPs (Fig. 3c). Only 14.4% of MNPs colocalized with early endosomes at 0 h, declining to 6.9% at 4 h and 6.2% at 24 h (Fig. 3d), similar to the colocalization of straight liposomes with early endosomes, 17.7%, 8.4%, and 6.1% at 0 h, 4 h, and 24 h, respectively (ESI Fig. S6a†). These results demonstrate that only a small fraction of fusogenic MNPs undergo endocytosis, while most of them bypass the endosomal pathway through liposome-membrane fusion.

We also examined plasma membrane localization of liposomes and MNPs as measured by colocalization of DiI-labeled liposomes and CellLight® plasma membrane-targeting GFP to the myristoylation/palmitoylation sequence from Lck tyrosine kinase. A decrease in plasma membrane localization was observed between 0 h and 4 h after liposome incubation, suggesting the fusion of liposomes with the plasma membrane (ESI Fig. S7†), in accordance with previous studies.^31^ To quantify distribution of MNPs to lysosomes, ARPE-19 cells were labeled with CellLight® lysosome-targeting Lamp1-GFP. Confocal imaging at 0 h, 4 h, and 24 h after liposome-coated MNPs incubation (Fig. 3e) showed an increased lysosome localization of MNPs from 9.2% to 45.8% to 58.8%, respectively (Fig. 3f). Lysosome targeting was also found for liposomes, indicated by the fraction of DiI-labeled liposomes localization to lysosome-targeted GFP, increasing from 9.8% at 0 h to 61.6% and 65.8% at 4 h and 24 h after liposome incubation (ESI Fig. S6b†). These results implicate lysosomes as an important targeting organelle for MNPs and liposome metabolism.

**Fig. 4:**
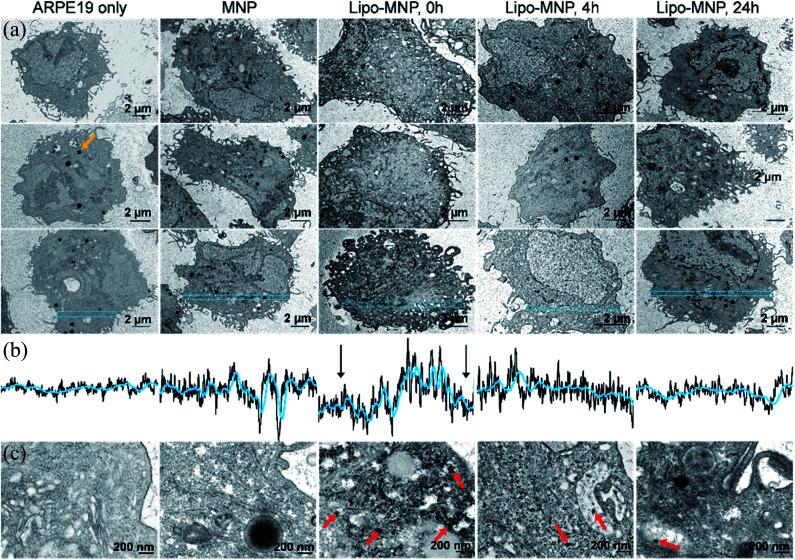
Distribution of MNPs in ARPE-19 cells. (a) Representative low magnification TEM images of ARPE-19 cells, ARPE-19 cells right after treatment with MNPs, and ARPE-19 cells at 0, 4, and 24 hours after treatment with liposome-coated MNPs (Lipo-MNP). Orange arrow points out a representative pigment granule in the ARPE-19 cells. (b) Line plots of normalized gray values in the area highlighted in blue from (a). The black arrows in the middle column indicate the drop of gray value, due to enhanced MNP delivery by liposome coating. (c) High magnification TEM images show the distribution of MNPs in cells. Both untreated ARPE-19 cells and ARPE-19 cells treated with MNPs showed show no obvious individual MNPs with a diameter around 50 nm. ARPE-19 cells treated with Lipo-MNPs showed many individual MNPs intracellularly, exemplified with red arrows. At 0 hours after lipo-MNP treatment, many individual MNPs were present in cells, close to the plasma membrane. These MNPs were not surrounded by a membrane structure, which indicates successful bypassing of endocytosis. At 4 hours after lipo-MNP treatment, MNPs were detected in both cytosol and lysosomes. At 24 hours after lipo-MNP treatment, there were much less MNPs in the cytosol compared to 0 hours, likely due to the lysosomal metabolism of MNPs.

MNP localization to cytosol suggested by fluorescence microscopy was confirmed with TEM imaging (Fig. 4). Of note, we were careful to distinguish MNPs from the dense black pigmented granules characteristic for mature ARPE-19 cells (*e.g.,* orange arrow in Fig. 4a). Although MNPs were hardly visible in low magnification TEM images due to their small size, their presence was indicated by the differing gray value pattern in the cytosol. Immediately after treatment with liposome-coated MNPs, APRE19 cells showed a decreased gray value along the cell membrane compared to the center of cell cytosol (Fig. 4b), which was likely due to the presence of MNPs along the cell membrane. This pattern was not seen in untreated or bare-MNP-treated cells because of the absence or limited uptake of MNPs. The gray value pattern disappeared after 4- and 24 hour treatments with liposome-coated MNPs, possibly due to diffusion and metabolism of MNPs in the cytosol. Representative high magnification TEM images also showed accumulation of MNPs close to the cell membrane right after treatment with liposome-coated MNP and dispersion of MNPs in the cytosol at 4 and 24 hours after treatment (Fig. 4c). Additionally, MNPs delivered by liposomes were obviously located in the cytosol directly without a membrane structure surrounding them, while MNPs without liposome coatings were surrounded, as an aggregate, by a membrane structure, which might indicate endocytosis of the MNPs aggregates (Fig. 4c).

**Fig. 5:**
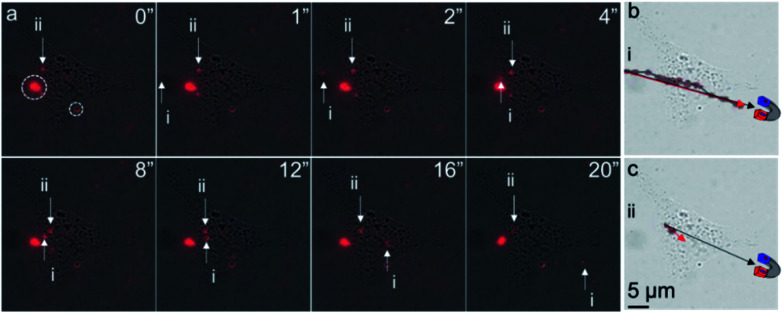
Motion tracking of intracellular and extracellular MNPs. (a) Location of MNPs (red) over time. Both extracellular MNP (i) and intracellular MNP (ii) were found to shift with an external magnet on the bottom left of the cells. Immobilized MNPs (dotted circles) were also found along the cell membrane. Motion tracking of (b) extracellular MNP (i) and (c) intracellular MNP (ii) over 20 seconds. The black and red arrows indicate the magnetically directed path of MNP motion. Unlike extracellular MNP, the moving path of the intracellular MNP was confined within the cell. All images share the same scale bar in (c). The cartoon magnets illustrate the proximate location of a neodymium magnet.

Finally, to determine whether intracellular MNPs could be attracted to and moved by an external magnet, live-cell imaging of the liposome-coated MNP-treated ARPE-19 cells was performed before and after the application of an external magnetic field. We used a neodymium magnet to apply a magnetic field of approximately 2000 Gauss to the cells. We observed three different motion responses upon magnetic field induction (Fig. 5). First, extracellular free MNPs showed a magnet-directed movement path (Fig. 5a and b), with a velocity of 4 μm s^−1^ in water that then decreased as the MNP moved over the cell. Second, we observed completely confined MNPs along the cell membrane, circled in Fig. 5a. These MNPs were likely bound during delivery and immobilized by the cell membrane. Third, some intracellular MNPs exhibited limited movement within the cell with a velocity of 0.325 μm s^−1^ in the first 8 seconds, after which they stopped moving towards the magnet (Fig. 5a). The motion path of these intracellular MNPs diverged approximately 15° from the magnet direction path (Fig. 5c). We hypothesize that this motion was limited by intracellular structures.

The lower velocity of the intracellular MNPs compared to extracellular MNPs suggested a higher cytoplasm viscosity than water. The viscosity of cytoplasm of ARPE-19 cells could be estimated according to the Stoke's law (*F* = 6π*μRv*, where *F*, *μ*, *R*, and *v* are the Stokes' drag force, viscosity of surrounding media, radius of the particle, and velocity of the particle).^13^ In Fig. 5a, it is fair to assume the particles i and ii had similar size and Stokes' drag force. The viscosity of water at 20 °C is 1.002 cP, so the calculated viscosity of the cytoplasm of ARPE-19 cells was approximately 12.33 cPa, which was in the range of reported intracellular viscosity in the cytoplasm of epithelial MDCK cells and Swiss 3T3 fibroblasts.^22^ Of note, using MNPs with uniform size and well-defined shape instead of the MNPs used in this work could provide more accurate calculation of the intracellular viscosity.

## Conclusions

In summary, MNPs were delivered into the cytosol of ARPE-19 cells directly with a novel fusogenic single-layer liposome coating method. This endocytosis-bypassing delivery route was validated with both confocal microscopy and TEM. Our findings revealed limited early endosome localization of MNPs, suggesting a successful bypass of endocytosis after liposome coating. In addition, the elevated portion of free MNPs and absence of membrane structure surrounding delivered MNPs in the cytosol indicates dissociation of MNPs from liposomes after cytoplasmic entry. The localization of liposome-coated MNPs with lysosomes was surprising. As critical destinations for the secretory, endocytic, and autophagic pathways,^36^ lysosomes are responsible for protein degradation and recycling. The exclusion of endocytic pathways makes the lysosome targeting of MNPs more interesting. By monitoring the intracellular motion of delivered MNPs, we were able to evaluate the viscosity of cytosol in ARPE-19 cells.

Free, extracellular MNPs usually undergo endocytosis followed by lysosomal degradation, which limits the direct interactions between MNPs and organelles. MNPs delivered to cytosol directly have great potential as an approach to facilitate interaction with specific organelles, which merits exploration in future experiments to investigate the effects of organelle localization on cellular functions.^13,37^ The endocytosis-bypassing delivery of MNPs provides a novel and potentially useful tool for subcellular magnetic localization and manipulation of organelles in living cells and tissues.

## Author contributions

F. C., M. B., D. M. and J. L. G. designed, and F. C. and M. B. performed the experiments and wrote the paper. M. N. assisted the cell studies and edited the language of the manuscript. D. M. and J. L. G. supervised this work and revised the manuscript.

## Conflicts of interest

There are no conflicts to declare.

## Supplementary Material

RA-011-D1RA03094A-s001

## References

[cit1] Jokerst J. V., Gambhir S. S. (2011). Acc. Chem. Res..

[cit2] Chen F., Si P., de la Zerda A., Jokerst J. V., Myung D. (2021). Biomater. Sci..

[cit3] Smith B. R., Gambhir S. S. (2017). Chem. Rev..

[cit4] Chen F., Hableel G., Zhao E. R., Jokerst J. V. (2018). J. Colloid Interface Sci..

[cit5] Cardoso V. F., Francesko A., Ribeiro C., Banobre-Lopez M., Martins P., Lanceros-Mendez S. (2018). Adv. Healthcare Mater..

[cit6] Lee J. E., Lee N., Kim H., Kim J., Choi S. H., Kim J. H., Kim T., Song I. C., Park S. P., Moon W. K., Hyeon T. (2010). J. Am. Chem. Soc..

[cit7] Theruvath A. J., Nejadnik H., Lenkov O., Yerneni K., Li K., Kuntz L., Wolterman C., Tuebel J., Burgkart R., Liang T., Felt S., Daldrup-Link H. E. (2019). Radiology.

[cit8] Lemaster J. E., Chen F., Kim T., Hariri A., Jokerst J. V. (2018). ACS Appl. Nano Mater..

[cit9] Gupta A. K., Naregalkar R. R., Vaidya V. D., Gupta M. (2007). Nanomedicine.

[cit10] Hou Z., Liu Y., Xu J., Zhu J. (2020). Nanoscale.

[cit11] Dobson J. (2006). Drug Dev. Res..

[cit12] Gan Q., Lu X., Yuan Y., Qian J., Zhou H., Lu X., Shi J., Liu C. (2011). Biomaterials.

[cit13] Steketee M. B., Moysidis S. N., Jin X. L., Weinstein J. E., Pita-Thomas W., Raju H. B., Iqbal S., Goldberg J. L. (2011). Proc. Natl. Acad. Sci. U. S. A..

[cit14] Cheng K., Li T.-S., Malliaras K., Davis D. R., Zhang Y., Marbán E. (2010). Circulation Research.

[cit15] Ito A., Shinkai M., Honda H., Kobayashi T. (2005). J. Biosci. Bioeng..

[cit16] Chen F., Zhao E. R., Hableel G., Hu T., Kim T., Li J., Gonzalez-Pech N. I., Cheng D. J., Lemaster J. E., Xie Y., Grassian V. H., Sen G. L., Jokerst J. V. (2019). ACS Nano.

[cit17] Cheng K., Shen D., Hensley M. T., Middleton R., Sun B., Liu W., De Couto G., Marban E. (2014). Nat. Commun..

[cit18] Kamei G., Kobayashi T., Ohkawa S., Kongcharoensombat W., Adachi N., Takazawa K., Shibuya H., Deie M., Hattori K., Goldberg J. L., Ochi M. (2013). The American Journal of Sports Medicine.

[cit19] Moysidis S. N., Alvarez-Delfin K., Peschansky V. J., Salero E., Weisman A. D., Bartakova A., Raffa G. A., Merkhofer R. M., Kador K. E., Kunzevitzky N. J., Goldberg J. L. (2015). Nanomedicine.

[cit20] Bartakova A., Kuzmenko O., Alvarez-Delfin K., Kunzevitzky N. J., Goldberg J. L. (2018). Investigative Ophthalmology & Visual Science.

[cit21] Xia X., Atkins M., Dalal R., Kuzmenko O., Chang K.-C., Sun C. B., Benatti C. A., Rak D. J., Nahmou M., Kunzevitzky N. J., Goldberg J. L. (2019). Investigative Ophthalmology & Visual Science.

[cit22] Puchkov E. (2013). Biochemistry (Moscow) Supplement Series A: Membrane and Cell Biology.

[cit23] Chen F., Ma M., Wang J., Wang F., Chern S. X., Zhao E. R., Jhunjhunwala A., Darmadi S., Chen H., Jokerst J. V. (2017). Nanoscale.

[cit24] Kim T., Lemaster J. E., Chen F., Li J., Jokerst J. V. (2017). ACS Nano.

[cit25] Doherty G. J., McMahon H. T. (2009). Annu. Rev. Biochem..

[cit26] Smith S. A., Selby L. I., Johnston A. P. R., Such G. K. (2019). Bioconjugate Chem..

[cit27] Krpetić Ž., Saleemi S., Prior I. A., Sée V., Qureshi R., Brust M. (2011). ACS Nano.

[cit28] Berry C. C., Curtis A. S. G. (2003). J. Phys. D: Appl. Phys..

[cit29] Du S., Liew S. S., Li L., Yao S. Q. (2018). J. Am. Chem. Soc..

[cit30] Kube S., Hersch N., Naumovska E., Gensch T., Hendriks J., Franzen A., Landvogt L., Siebrasse J.-P., Kubitscheck U., Hoffmann B., Merkel R., Csiszár A. (2017). Langmuir.

[cit31] Kim B., Pang H.-B., Kang J., Park J.-H., Ruoslahti E., Sailor M. J. (2018). Nat. Commun..

[cit32] Yoshimoto M. (2017). Methods Mol. Biol..

[cit33] Clement J. H., Schwalbe M., Buske N., Wagner K., Schnabelrauch M., Görnert P., Kliche K. O., Pachmann K., Weitschies W., Höffken K. (2006). J. Cancer Res. Clin. Oncol..

[cit34] Urbano-Bojorge A. L., Casanova-Carvajal O., Félix-González N., Fernández L., Madurga R., Sánchez-Cabezas S., Aznar E., Ramos M., Serrano-Olmedo J. J. (2018). Nanotechnology.

[cit35] Demchenko A. P. (2020). Methods Appl. Fluoresc..

[cit36] Ferguson S. M. (2019). Neurosci. Lett..

[cit37] Pita-Thomas W., Steketee M. B., Moysidis S. N., Thakor K., Hampton B., Goldberg J. L. (2015). Nanomedicine.

