# Supplementary material for: Fusogenic liposome-enhanced cytosolic delivery of magnetic nanoparticles
Source: RSC Adv. 2021 Nov 4;11(57):35796–805. doi: 10.1039/d1ra03094a (PMC9043121; doi:10.1039/d1ra03094a)
Supplement: RA-011-D1RA03094A-s001 [file RA-011-D1RA03094A-s001.pdf]

Electronic Supplementary Material (ESI) for RSC Advances.
This journal is © The Royal Society of Chemistry 2021

Fusogenic Liposome-Enhanced Cytosolic Delivery of Magnetic Nanoparticles

Fang Chen[1, 2] #, Minjuan Bian[1] #, Michael Nahmou[1], David Myung[1, 2, 3, *], and Jeffrey L Goldberg[1, 2, *]

[1] Department of Ophthalmology, Byers Eye Institute at Stanford University, Palo Alto, CA, 94304, USA;
[2] VA Palo Alto Health Care System, Palo Alto, CA, 94304, USA;
[3] Department of Chemical Engineering, Stanford University, Stanford, CA, 94305, USA
# These authors contributed equally.

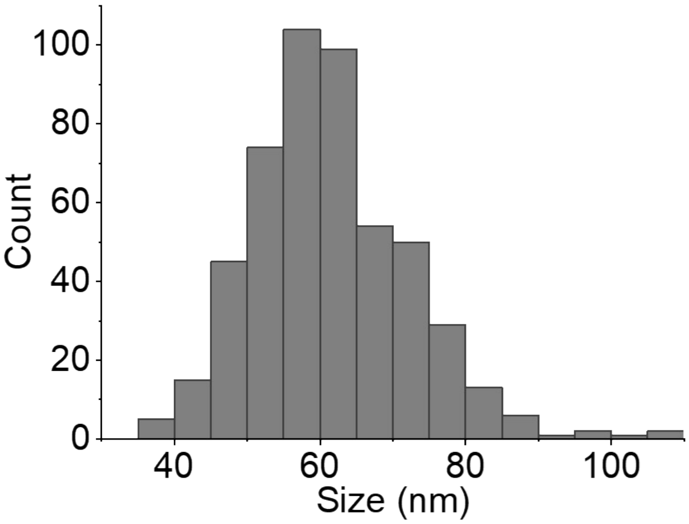

Figure S1. Size distribution (diameter) of the magnetic nanoparticles based on TEM measurements. The average diameter of the magnetic nanoparticles is 61 nm, and standard deviation is 11 nm (n=500).

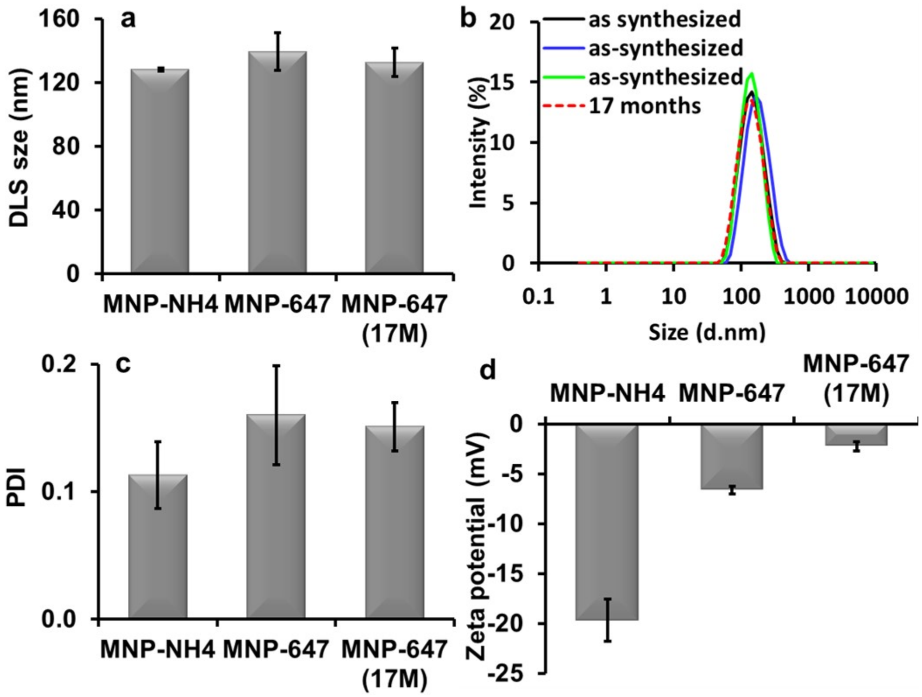

Figure S2. Dye conjugation and stability of magnetic nanoparticles. (a) Dynamic light scattering size of magnetic nanoparticles before (MNP-NH4) and after (MNP-647) dye conjugation, and dye-conjugated MNPs after storage in dark at 4°C for 17 months (MNP-647(17M)). (b) Size distribution of magnetic nanoparticles after dye conjugation and 17-month storage. (c) Polydispersity index of magnetic nanoparticles. (d) Surface zeta potential of magnetic nanoparticles.

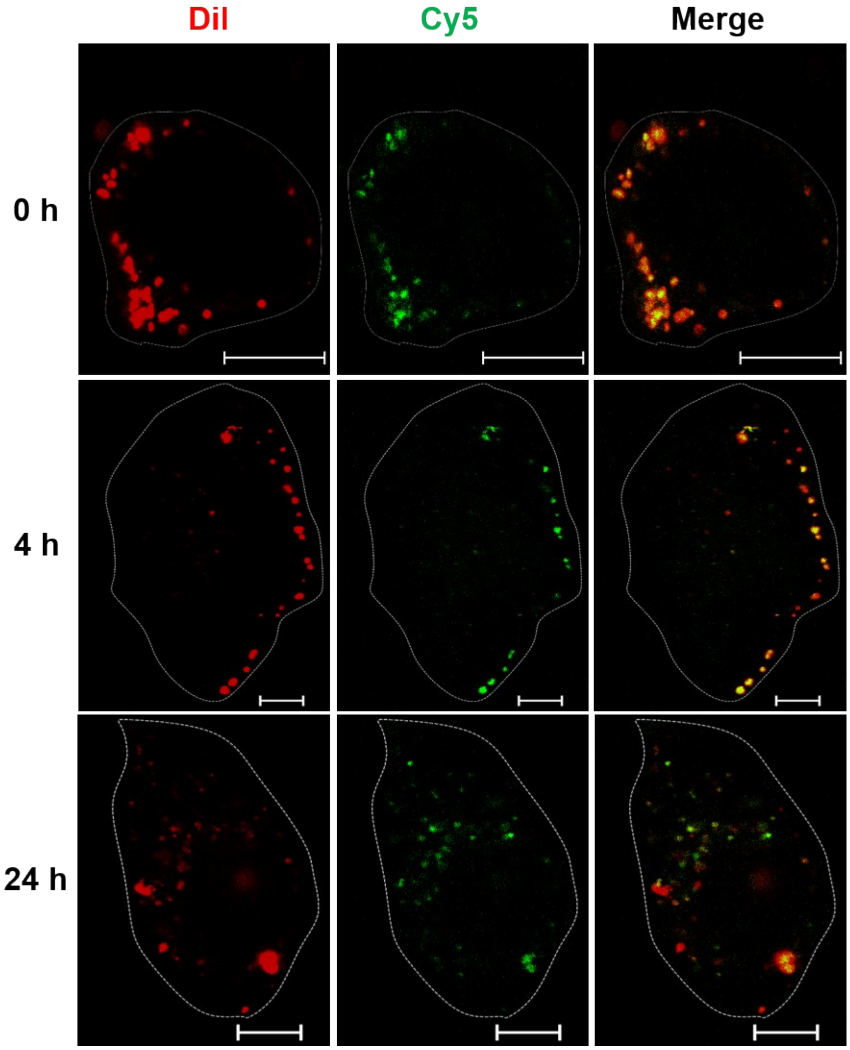

Figure S3. Co-localization of DiI and Cy5 signals from liposomes after incubation with ARPE-19 cells. Liposomes labeled with 1,2-distearoyl-sn-glycero-3-phosphoethanolamine-N-[amino(polyethylene glycol)-2000]-N-(Cyanine 5) (DSPE-PEG-CY5) and DiI were incubated with ARPE-19 cells for 10 min. Cells were fixed with 4% paraformaldehyde (PFA) for 10 min at room temperature at 0 h, 4 h and 24 h after liposome incubation. DiI and Cy5 signals were acquired by confocal microscopy. Scale bar = 10 µm.

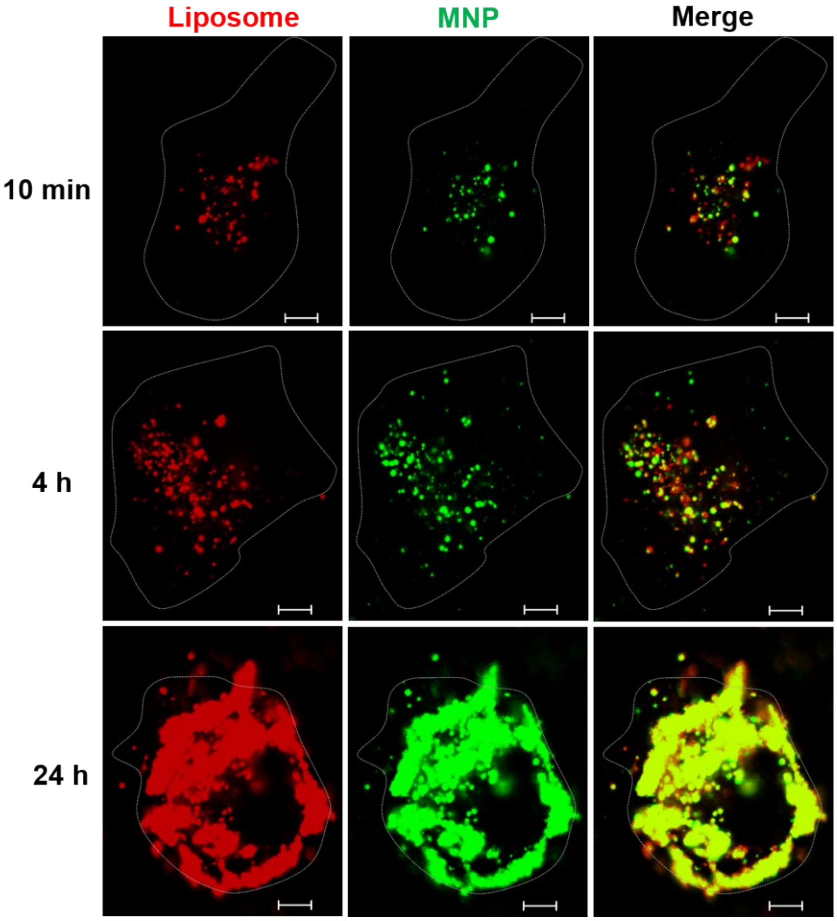

Figure S4. Administration of liposome-coated MNPs with different incubation times in ARPE-19 cells. Liposome-coated MNPs suspended in cell culture medium with MNPs at a concentration of 100 µg/ml were administrated to ARPE-19 cells for 10 min, 4 h or 24 h. Cells were washed with PBS and fixed with 4% PFA for 10 min at room temperature at 24 h after liposome application. DiI and Alexa Fluor 647 signals were acquired by confocal microscopy. Scale bar = 10 µm.

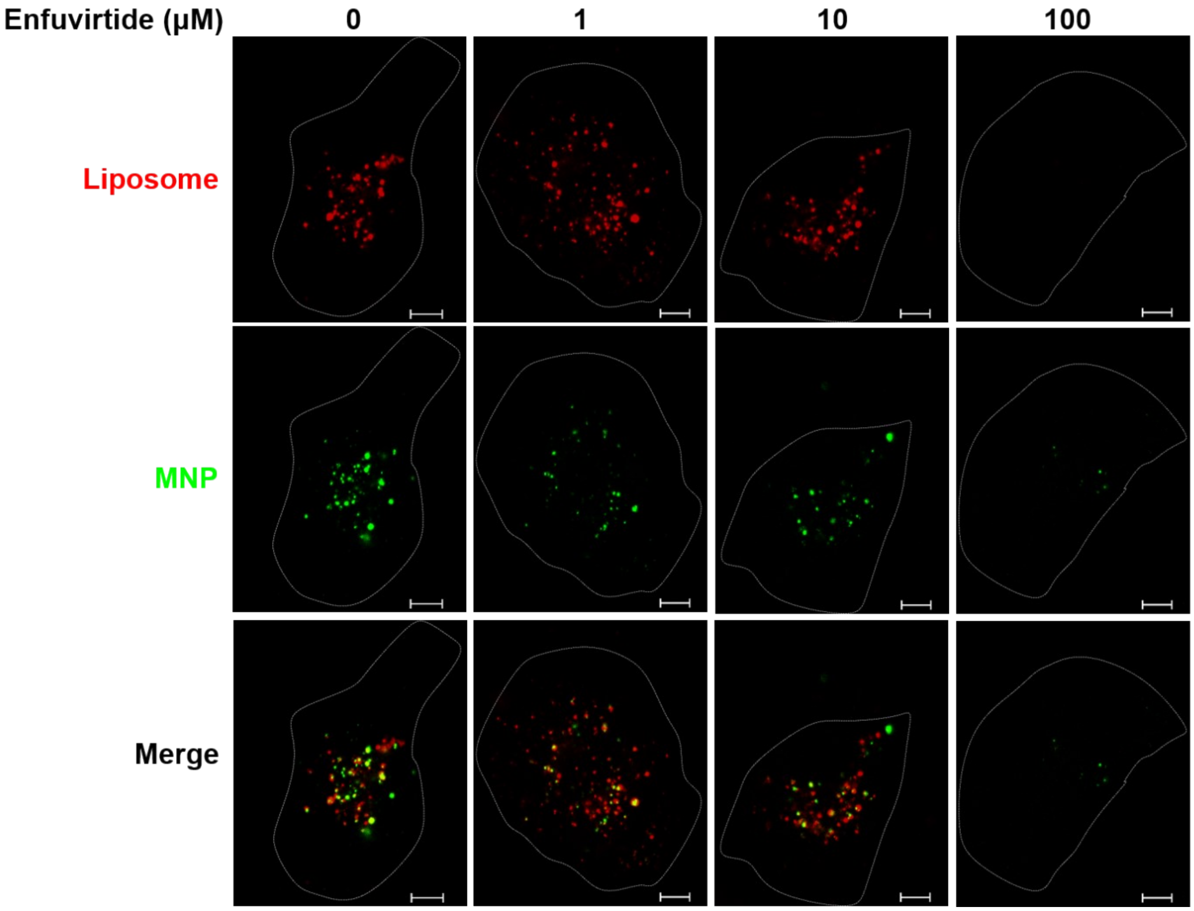

Figure S5. The effect of membrane fusion inhibitor on the uptake of liposome-coated MNPs in ARPE-19 cells. ARPE-19 cells were treated with membrane fusion inhibitor enfuvirtide at 1 µM, 10 µM and 100 µM for 1 h prior to 10 min incubation of liposome coated MNPs administration. Cells were washed with PBS and fixed with 4% PFA for 10 min at room temperature at 24 h after liposome incubation. DiI and Alexa Fluor 647 signals were acquired by confocal microscopy. Scale bar = 10 µm.

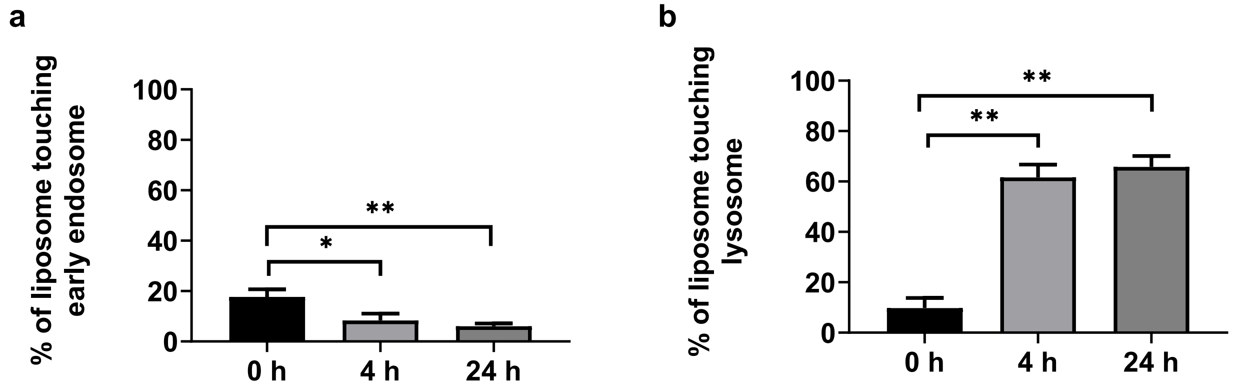

Figure S6. Intracellular distribution of liposome after incubation with ARPE-19 cells. CellLight® early endosome-GFP targeting Rab5a or CellLight® lysosome-GFP targeting Lamp1 were incubated with ARPE-19 for 24 h, DiI labeled liposome-coated MNPs were then administrated to ARPE-19 cells for 10 min respectively. Cells were washed with PBS and fixed with 4% PFA for 10 min at room temperature at 0 h, 4 h and 24 h after incubation with liposome-coated MNPs. Early endosome-GFP and DiI or lysosome-GFP and DiI signals were acquired by confocal microscopy; 10-15 cells from each group were recorded. Measurement of identified early endosome-GFP and DiI or lysosome-GFP and DiI positive objects were collected by Volocity, and the fractions of DiI signals touching early endosome-GFP(a) or lysosome-GFP(b) were calculated and analyzed by one-way ANOVA followed by Tukey's multiple comparisons test (* $p<0.05$, ** $p<0.01$, $n = 10$-15 cells).

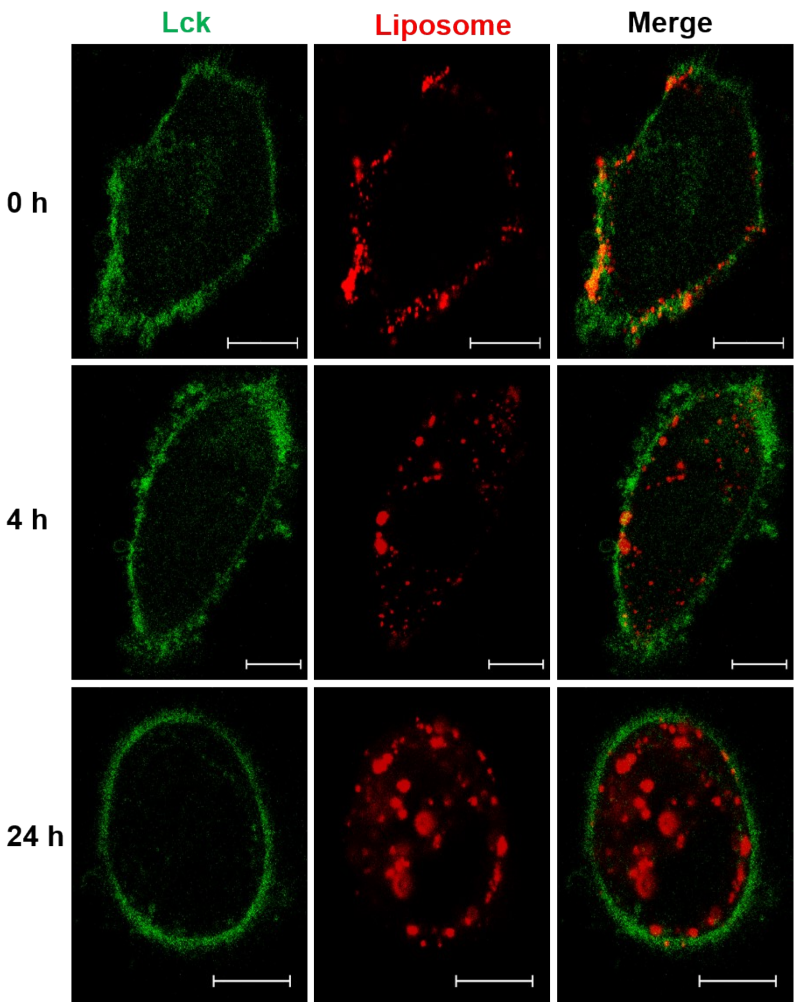

Figure S7. Co-localization of liposomes with Lck after incubation with ARPE-19. CellLight® plasma membrane-GFP targeting the myristolyation/palmitoylation sequence from Lck tyrosine kinase was incubated with ARPE-19 for 24 h, DiI labeled liposomes were then administrated to ARPE-19 for 10 min. Cells were washed with PBS and fixed with 4% PFA for 10 min at room temperature at 0 h, 4 h and 24 h after liposome incubation. Lck and DiI signals were acquired by confocal microscopy. Scale bar = 10 µm.
